# Supplementary material for: The interactome of intact mitochondria by cross-linking mass spectrometry provides evidence for coexisting respiratory supercomplexes
Source: Mol Cell Proteomics. 2017 Dec 8;17(2):216–32. doi: 10.1074/mcp.RA117.000470 (PMC5795388; doi:10.1074/mcp.RA117.000470)
Supplement: Supplemental Data [file supp_RA117.000470_133922_0_supp_23978_fzffwf.zip › spectra_annotation/mito_spectra_annotation/260-1-2-1-6-1.pdf]

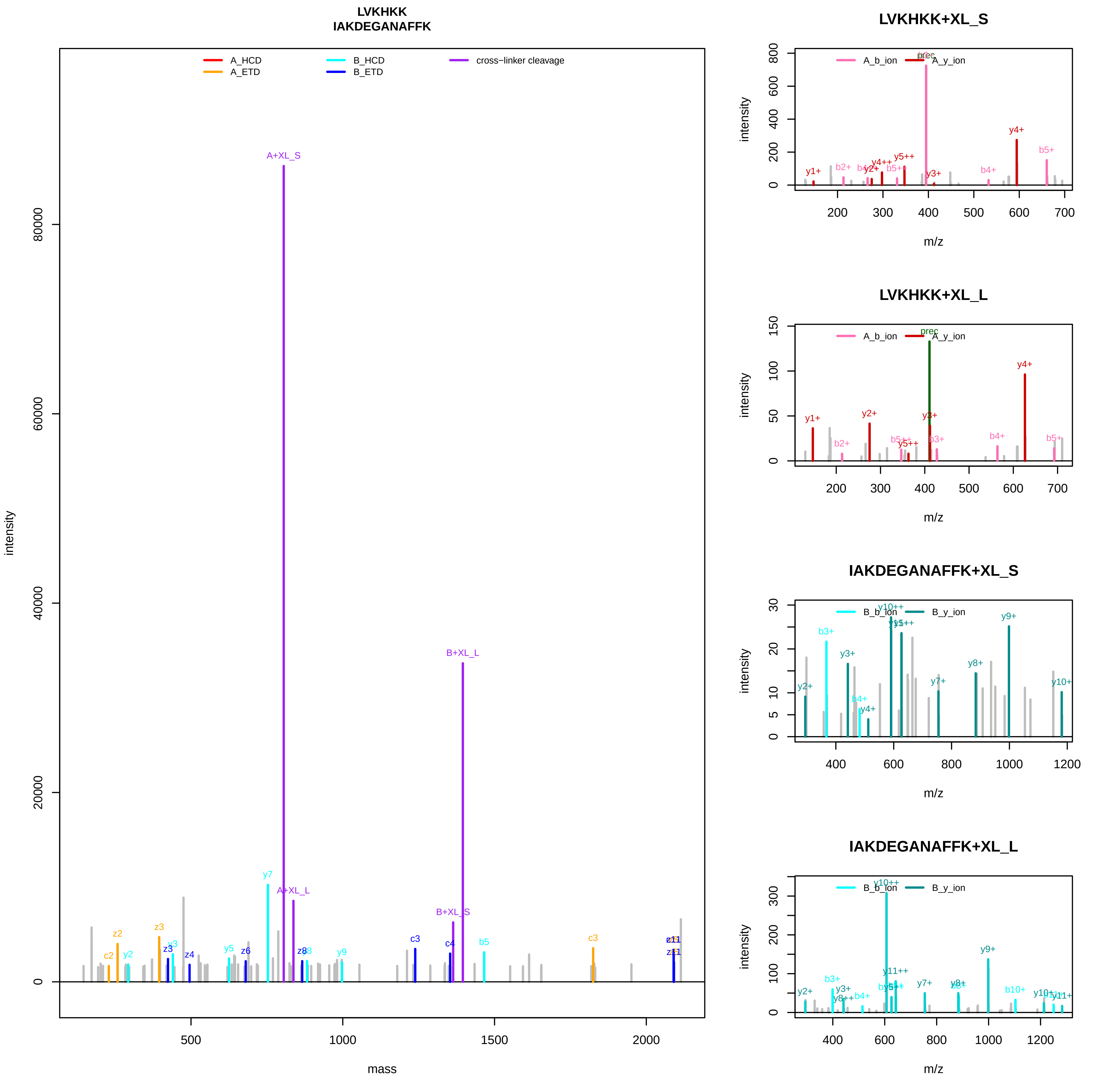

LVKHKK
IAKDEGANAFFK
A_HCD
A_ETD
B_HCD
B_ETD
cross−linker cleavage
A+XL_S
B+XL_L
A+XL_L
B+XL_S
intensity
mass
0
20000
40000
60000
80000
500
1000
1500
2000
c2
z2
y2
z3
z3
y3
z4
y5
z6
y7
z8
y8
y9
c3
c4
b5
c3
c11
z11
LVKHKK+XL_S
A_b_ion
A_y_ion
prec
y1+
b2+
y2+
y4++
y5++
b5++
y3+
b4+
y4+
b5+
m/z
LVKHKK+XL_L
y1+
b2+
y2+
b5++
y5++
y3+
b3+
b4+
y4+
b5+
IAKDEGANAFFK+XL_S
B_b_ion
B_y_ion
y2+
b3+
y3+
b4+
y4+
y10++
y11++
y7+
y8+
y9+
y10+
IAKDEGANAFFK+XL_L
y2+
b3+
y3+
y8++
b4+
y10++
y11++
y7+
y8+
y9+
b10+
y10+
y11+
